# How the brain stays in sync with the real world

**DOI:** 10.7554/eLife.85301

**Published:** 2023-01-19

**Authors:** Damian Koevoet, Andre Sahakian, Samson Chota

**Affiliations:** 1 Experimental Psychology, Helmholtz Institute, Utrecht University Utrecht Netherlands

**Keywords:** EEG, prediction, motion, neural delays, latency, brain, Human

## Abstract

The brain can predict the location of a moving object to compensate for the delays caused by the processing of neural signals.

**Related research article** Johnson PA, Blom T, van Gaal S, Feuerriegel D, Bode S, Hogendoorn H. 2023. Position representations of moving objects align with real-time position in the early visual response. *eLife* **12**:e82424. doi: 10.7554/eLife.82424.

In professional baseball the batter has to hit a ball that can be travelling as fast as 170 kilometers per hour. Part of the challenge is that the batter only has access to outdated information: it takes the brain about 80–100 milliseconds to process visual information, during which time the baseball will have moved about 4.5 meters closer to the batter (Allison et al., 1994; Thorpe et al., 1996). This should make it virtually impossible to consistently hit the baseball, but the batters in Major League Baseball manage to do so about 90% of the time. How is this possible?

Fortunately, baseballs and other objects in our world are governed by the laws of physics, so it is usually possible to predict their trajectories. It has been proposed that the brain can work out where a moving object is in almost real time by exploiting this predictability to compensate for the delays caused by processing (Hogendoorn and Burkitt, 2019; Kiebel et al., 2008; Nijhawan, 1994). However, it has not been clear how the brain might be able to do this.

Since predictions must be made within a matter of milliseconds, highly time-sensitive methods are needed to study this process. Previous experiments were unsuccessful in determining the exact timing of brain activity (Wang et al., 2014). Now, in eLife, Philippa Anne Johnson and colleagues at the University of Melbourne and the University of Amsterdam report new insights into motion processing (Johnson et al., 2023).

Johnson et al. used a combination of electroencephalogram (EEG) recordings and pattern recognition algorithms to investigate how long it took participants to process the location of objects that either flashed in one place (static objects) or moved in a straight line (moving objects). Using machine learning techniques, Johnson et al. first identified how the brain represents a non-moving object (Grootswagers et al., 2017). They accurately mapped patterns of neural activity, which corresponded to the location of the static object during the experiment. Participants took about 80 milliseconds to process this information (Figure 1).

**Figure 1.**
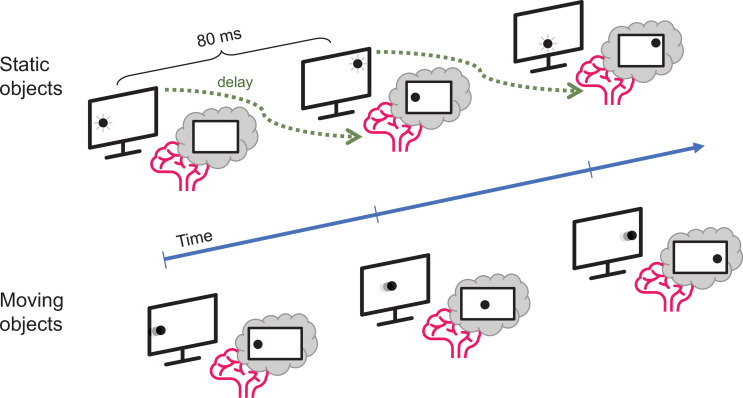
Motion processing in the human brain. Johnson et al. compared how long it takes the brain to process visual information about static objects and moving objects. The static objects (top) did not move but were briefly shown in unpredictable locations on the screen: the delay between the appearance of the object and the representation of its location in the brain was about 80 milliseconds. However, when the object moved in a predictable manner (bottom), the delay was much smaller.

Strikingly, Johnson et al. discovered that the brain represented the moving object at location different to where one would expect it to be (i.e., not at the location from 80ms ago). Instead, the internal representation of the moving object was aligned to its actual current location so that the brain was able to track moving objects in real time. The visual system must therefore be able to correct the position by at least 80 milliseconds worth of movement, indicating that the brain can effectively compensate for temporal processing delays by predicting (or extrapolating) where a moving object will be located in the future.

To fully grasp how motion prediction processes compensate for the lag between the external world and the brain, it is important to know where in the visual system this compensatory mechanism occurs. Johnson et al. showed that the delay was already fully compensated for in the visual cortex, indicating that the compensation happens early during visual processing. There is evidence to suggest that some degree of motion prediction occurs in the retina, but Johnson et al. argue that this on its own is not enough to fully compensate for the delays caused by neural processing (Berry et al., 1999).

Another possibility is that a brain area involved in a later stage of motion perception, called the middle temporal area, may also play a role in predicting the location of an object (Maus et al., 2013). This region is thought to provide predictive feedback signals that help to compensate for the neural processing delay between the real world and the brain (Hogendoorn and Burkitt, 2019). More research is needed to test this theory, for example, by directly recording neurons in the middle temporal area in primates and rodents using intracranial electrodes. Gaining access to such accurate spatial and temporal neural information might be key to identifying where predictions are made and what they foresee exactly.

The work of Johnson et al. confirms that motion prediction of around 80–100 milliseconds can almost completely compensate for the lag between events in the real world and their internal representation in the brain. As such, humans are able to react to incredibly fast events – if they are predictable, like a baseball thrown at a batter. Neural delays need to be accounted for in all types of information processing within the brain, including the planning and execution of movements. A deeper understanding of such compensatory processes will ultimately help us to understand how the human brain can cope with a fast world, while the speed of its internal signaling is limited. The evidence here seems to suggest that we overcome these neural delays during motion perception by living in our brain’s prediction of the present.
